# Effects of Processing Conditions and Simulated Digestion In Vitro on the Antioxidant Activity, Inhibition of Xanthine Oxidase and Bioaccessibility of Epicatechin Gallate

**DOI:** 10.3390/foods12142807

**Published:** 2023-07-24

**Authors:** Miao Zhu, Xiaoyun Fei, Deming Gong, Guowen Zhang

**Affiliations:** State Key Laboratory of Food Science and Technology, Nanchang University, Nanchang 330047, China

**Keywords:** epicatechin gallate, xanthine oxidase, inhibitory mechanism, food processing conditions, simulated digestion in vitro

## Abstract

The bioactivity and gastrointestinal stability of epicatechin gallate (ECG) may be affected by processing conditions. Results showed that the antioxidant ability and inhibitory activity on xanthine oxidase (XO) of ECG were higher at low pH values. Appropriate microwave and heating treatments improved the antioxidant (the scavenging rate increased from 71.75% to 92.71% and 80.88% under the microwave and heating treatments) and XO inhibitory activity (the inhibitory rate increased from 47.11% to 56.89% and 51.85% at the microwave and heating treatments) of ECG. The treated ECG led to a more compact structure of XO. Moreover, there may be synergistic antioxidant and inhibitory effects between ECG and its degradation products. The bioaccessibility of ECG after simulated digestion was untreated > microwave > heating, and the microwave−treated ECG still had good XO inhibitory activity after digestion. These findings may provide some significant information for the development of functional foods enriched in catechins.

## 1. Introduction

**Figure 1:**
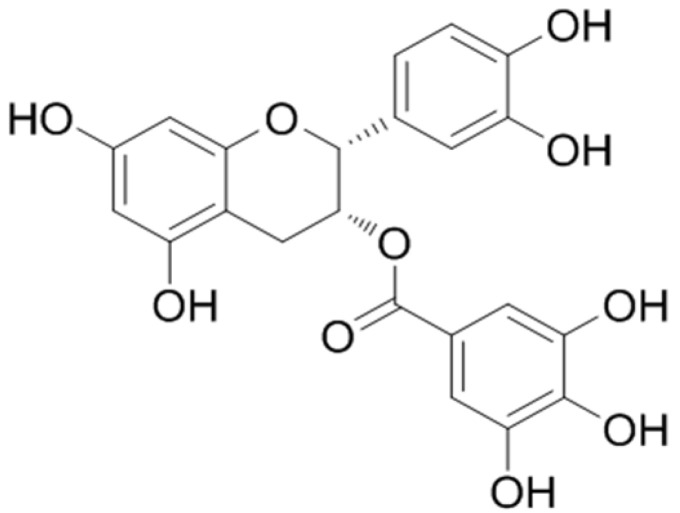
The molecular structure of epicatechin gallate (ECG).

Catechin is the main tea polyphenol (accounting for 60–80%), and is widely used in food, cosmetics and medicine [1]. Epicatechin gallate (ECG, the structural formula is shown as Figure 1), a typical ester-type catechin, has recently attracted much attention due to its beneficial effects on human health. Compared with other catechins, ECG not only has obvious common characteristics of catechins, but also has many unique physiological effects due to its higher number of phenolic hydroxyl groups and smaller steric hindrance, such as scavenging free radicals, lowering uric acid, anti-mutation, anti-inflammatory, hypoglycemic, anti-obesity and enhancing immunity activities [2]. It was found that ECG effectively prevented the oxidative stress and improved glucose, lipid and uric acid metabolism [3]. Nowadays, as a green and healthy natural product, ECG has been used in the development of beverages, grains, oils and functional foods, and has great development potential in the food industry.

Xanthine oxidase (XO) is a molybdenum-containing flavoenzyme and mainly responsible for regulating catabolism of purine in human body. Hypoxanthine can be catalyzed by XO to xanthine and then continuously oxidized to produce uric acid, accompanied by the formation of O_2_^−^ or H_2_O_2_ [4]. XO is often used as the main protein target for the prevention and treatment of hyperuricemia and gout, because its activity directly regulates the level of uric acid [5]. Most of the commonly used hypouricemic medicines in clinic may cause many patients to suffer from toxicity and side effects. For instance, many patients who took allopurinol experienced diarrhea, skin rash, liver lesion and bone marrow damage [6]. Therefore, some natural XO inhibitors derived from plants, such as polyphenols, flavonoids and triterpenes, are gradually praised by experts in the fields of medicine, nutrition and agricultural sciences due to their high safety, good efficacy and small adverse reactions. In our previous study [7], ECG was found to be an excellent mixed-type XO inhibitor, and reduced the production of free radicals by reducing XO molecule and inhibiting the formation of uric acid. Therefore, ECG has broad application prospects in inhibiting XO activity and scavenging free radicals.

At present, in order to meet the needs of consumers for the flavor, color, sensory properties and nutritional value of food, various processing technologies have been developed in the food industry, such as acidification, heating, fermentation, microwave and ultrasound [8,9]. However, these processing techniques may affect the stability, bioactivity and bioaccessibility of food components (proteins, lipids, polyphenols, etc.), thus altering the quality of food [10]. The research on the effects of processing conditions on the functional characteristics of food components has become a hot topic in the field of food nutrition and processing. Traditional heating is one of the most commonly used processing methods, which can improve food flavor and inhibit the growth of microorganisms. However, heating may also degrade and oxidize some nutritional elements, influence the constitution of natural ingredients, thus affecting the biological activity of food [11]. Yin et al. [12] found that heating significantly affected the content of functional components and antioxidant activity of Chinese herb candy, and the Maillard reaction product formed at high temperature increased the antioxidant capacity of the samples. Microwave treatment is an emerging food processing technology that is booming in home cooking and food industrial production with a series of advantages, such as high heating efficiency, short processing time, low operating cost and precise control [13]. Pérez-Grijalva et al. [14] reported that the contents of polyphenols and anthocyanin monomers in the final blackberry juice product were increased and the microbial growth was inhibited after microwave treatment prior to juice extraction, indicating the great potential of microwave in improving the nutritional value of blackberry juice. It was also reported that a combination of microwave and hot air treatment enhanced the inhibitory ability of polyphenols from *Asparagus* on tyrosinase, which provided a new approach for improving the anti-tyrosinase activity of phenols [15]. In addition, different pH conditions during the food processing also influenced the physicochemical properties of the components. Zheng et al. [16] explored the stability of a novel inhibitory peptide (GYGYNY) of angiotensin I-converting enzyme (ACE) and its inhibitory activity at different pH conditions, and found that GYGYNY was basically stable in the pH range of 3.0–11.0, and its inhibitory ability against ACE was weakest at pH 5.0. More and more attention has been paid to the research of the effects of processing conditions on the biological activity of natural ingredients related to health. ECG contains multiple phenolic hydroxyl groups, with highly exposed active sites, and is extremely sensitive to the conditions, such as temperature, acidity and metal ions, thus the oxidation, hydrolysis, isomerization and complexation reactions may occur during the processing of ECG, leading to the alterations in its biological activity, which may ultimately affect the flavor, color and functional quality of tea products [17]. However, to our knowledge, little has been known about the effects of these food processing treatment methods on ECG. Therefore, it is of great importance to explore the effects of common processing conditions on the stability and biological activity of ECG for the development of functional foods enriched catechins.

After food is ingested by human body, the active ingredients need to be digested by the gastrointestinal tract before they can further play their physiological functions. At present, the gastrointestinal digestive stability of food components has also been the focus of researchers in the field of food nutrition. As reported by Seczyk et al. [18], gastrointestinal digestion, food matrix and hydrothermal treatment had significant negative effects on the bioaccessibility and physiological activities of phenolic compounds. Marchese et al. [19] found that green tea polyphenols were relatively stable in simulated gastric fluid through the simulated digestion experiments in vitro, and the samples could maintain antibacterial activity after gastrointestinal digestion. It could be seen that the beneficial effects of bioactive ingredients are inseparable from the gastrointestinal digestion and fermentation. Therefore, the effects of gastrointestinal digestion on ECG need to be further investigated.

Therefore, the objective of this study was to analyze the effects of heating, microwave and pH conditions on the activities of ECG for scavenging free radical and inhibiting XO activity by multiple spectroscopic methods, and the possible degradation and transformation mechanism was also explored through HPLC analysis. A simulated digestion model in vitro was established to explore the changes in ECG in physiological activity and bioaccessibility. This study may offer the basic knowledge for the physiological functions of ECG in vivo, which is expected to promote the comprehensive utilization of catechins and the industrial production of catechin-rich foods.

## 2. Materials and Methods

### 2.1. Materials

ECG (purity ≥ 98%) was provided by Shanghai Yuanye Biotechnology Co., Ltd. (Shanghai, China), and was dissolved in 40% ethanol (*v*/*v*) to prepare the stock solution (5.0 mM). XO (from milk, Grade I, 10.87 units mL^−1^) and xanthine (purity ≥ 98%) were purchased from Beijing Solarbio Technology Co., Ltd. (Beijing, China), which were dissolved in Tris−HCl buffer (pH 7.4, 0.05 M) to prepare the stock solutions at concentrations of 5.0 μM and 0.5 mM, respectively. 2,2-Diphenyl-1-picrylhydrazyl (DPPH) from Sigma–Aldrich Chemical. (St. Louis, MO, USA) was prepared as a stock solution in absolute ethanol. α-Amylase (Analytical reagent, 50 U/mg), pepsin (Grade USP, 15 kU/mg), trypsin (Grade USP, 2.5 kU/mg) and bile salt (Biological reagent) were obtained from Sigma-Aldrich, Aladdin Biochemical Technology Co. (Shanghai, China), Shanghai Yuanye and Solarbio, respectively. Gallic acid (GA, purity > 99%) was purchased from Aladdin. Epigallocatechin (EGC, purity ≥ 99%), catechin gallate (CG, purity ≥ 99%) and epicatechin (EC, purity ≥ 99%) were provided by Shanghai Yuanye. All other chemical reagents were of chemical purity, and the ultrapure water was used in the study.

### 2.2. Different pH Conditions

According to the method of Chai et al. [20] with slight modification, the phosphate buffer solution was prepared, and then the pH value of the buffer solutions was adjusted to 2.0, 3.0, 4.0, 5.0, 6.0, 7.0, 7.4, 8.0, 9.0 and 10.0 by HCl and NaOH, respectively. ECG (5.0 mM) stock solution was separately dissolved in the above buffer solutions and incubated at room temperature for 30 min in the dark. Then the pH value of the sample solutions was adjusted to 7.0 for later analysis. The UV–vis absorption spectra of ECG samples after treatment were measured through an UV–vis spectrophotometer (UV–2450, Shimadzu, Tokyo, Japan).

### 2.3. Conventional Heating Treatment

The ECG solutions were prepared and heated in an electric thermostatic water bath (HH–6, Shanghai Tenlin Instrument Co., Ltd., Shanghai, China) at 25, 37, 60, 80 and 100 °C in the dark for 20, 40 and 60 min, respectively. Then the samples were quickly cooled in an ice water bath and placed in a refrigerator at –20 °C to terminate the reaction.

### 2.4. Microwave Heating Treatment

The ECG samples placed in the colorimeter tube were pretreated for 1, 3 and 6 min with the power of 160, 320, 480, 640 and 800 W, respectively, in a microwave oven (Galanz, G80F23CN1L−SD, Shunde, China). After heating, the ECG samples were cooled in ice water until the temperature dropped to room temperature. Solvents were then added to prepare ECG stock solutions and were frozen at –20 °C for storage.

### 2.5. Analysis of DPPH Free Radical Scavenging Activity

The antioxidant activity of ECG was evaluated with the method of Aalim et al. [21] with slight modifications. Firstly, DPPH was dissolved in ethanol to prepare a sample solution with a concentration of 0.15 mM, and then different concentrations of untreated, heated and microwaved ECG samples were added to the DPPH solution, respectively, and mixed to form a series of reaction solutions (where the concentrations of ECG were 0, 3.0, 6.0, 9.0, 12.0 and 15.0 μM, respectively). The mixed solutions were placed in a dark room for 30 min and waited for the reaction to proceed completely. The absorbance of the samples was measured at 517 nm using an enzyme-labeled instrument (Varioskan LUX, Thermo Fisher Scientific, Waltham, MA, USA). Absolute ethanol was used as a control, and the decreasing percentage in absorbance represented the DPPH radical scavenging rate of ECG.

### 2.6. Relative Activity Measurement of XO

The relative activity of XO was determined by the time-dynamics module of UV–vis spectrophotometer. The untreated, heated and microwaved ECG samples were evenly mixed with XO (0.075 μM) in Tris−HCl buffer, and then the mixed solutions were incubated at 37 °C for 30 min. Xanthine (50.0 μM) was added to initiate the reaction, and the absorbance values of the samples were measured at 293 nm within 200 s. The relative activity was determined by the following formula: the relative enzymatic activity (%) = *R*/*R*_0_ × 100, where *R* and *R*_0_ are the reaction rates of XO with and without of ECG, respectively.

### 2.7. Fluorescence Quenching and Thermodynamic Analysis

The effects of heating (100 °C, 20 min) and microwave (800 W, 6 min) treatments on the binding properties of ECG with XO were investigated using the method of Zhou et al. [22]. The excitation wavelength and the slit widths of excitation and emission for the fluorescence photometer (F–7000, Hitachi, Tokyo, Japan) were 280 nm and 2.5 nm, respectively, and the scanning range was 300−500 nm. The ECG samples (0–30.0 μM) were added to the XO solution (1 μM) successively, and the fluorescence spectra of the mixed samples were measured at 25, 31 and 37 °C, respectively. The obtained fluorescence data were subtracted the internal filter effect by the following equation [23]:

(1) $$F_{c}{=F}_{m}e^{{(A}_{1}{+A}_{2})/2}$$

where *F*_m_ and *F*_c_ are the measured and corrected fluorescence intensity values, respectively; *A*_1_ and *A*_2_ denote the absorbance values of ECG at excitation and emission wavelengths, respectively.

The following Stern–Volmer formula was used to calculate the quenching rate constant (*K*_sv_) of ECG and analyze the quenching mechanism [24]:

(2) $$\frac{F_{0}}{F}{=1+K}_{\mathrm{SV}}\left[Q\right]{=1+K}_{q}\tau_{0}\left[Q\right]$$

where *F* and *F*_0_ represent the fluorescence intensity of XO at the emission wavelength in the presence and absence of ECG, respectively; [Q] is the concentration of ECG; *τ*_0_ (10^−8^ s) is the average fluorescence lifetime of the biomacromolecules [25].

For the static fluorescence quenching, the following logarithmic equation was used to further obtain the values of binding constant (*K*_a_) and number of binding sites (*n*):

(3) $$\log\frac{F_{0}-F}{F}{=n\log K}_{a}-n\log\frac{1}{\left[Q_{t}\right]-\frac{{(F}_{0}-F)\left[P_{t}\right]}{F_{0}}}$$

where [*Q*_t_] and [*P*_t_] are the concentrations of ECG and XO, respectively.

In addition, the values of important thermodynamic parameters, such as entropy change (Δ*S*°), enthalpy change (Δ*H*°) and Gibbs free energy change (Δ*G*°) during the binding of ECG with XO were obtained from the classical van’t Hoff equation [26]:

(4) $${\log K}_{a}=-\frac{\Delta H{}^{\circ}}{2.303RT}+\frac{\Delta S{}^{\circ}}{2.303R}$$

(5) ΔG°=ΔH°−TΔS°

where *R* is the gas constant (8.314 J mol^−1^ K^−1^), and *T* represents the experimental temperature.

### 2.8. Measurements of Circular Dichromatic (CD) Spectra

The effects of two heating manners on the change in the secondary structure of XO by ECG were analyzed by CD spectroscopic experiments. The untreated, heated (100 °C, 20 min) and microwaved (800 W, 6 min) ECG at different concentrations were added into XO (1.0 μM) to prepare samples with the molar ratios ([ECG]/[XO]) of 0:1, 10:1, 20:1 and 40:1, respectively, then the solutions were incubated at room temperature for 30 min. The CD spectra of samples were measured by a CD spectrometer (Bio−Logic MOS 450, Claix, France) under constant nitrogen blowing, and the secondary structure contents of XO were calculated by an online program.

### 2.9. HPLC Analysis

The HPLC analysis of ECG was conducted using the method of Giusti et al. [27]. The samples and standards were dissolved in 40% ethanol and anhydrous ethanol, respectively. All samples were filtered through a 0.22 μm syringe nylon membrane. The initial concentration of ECG samples and the injection concentration of standards were both 1.0 mM, and each injection volume was 20 μL. An Agilent 1260 series HPLC (Agilent Technologies, Santa Clara, CA, USA) was used for the qualitative and quantitative analysis of ECG samples. The column and detector were Zorbax Eclipse XDB-C18 (5 μm, 4.6 mm × 250 mm) and diode-array detector (DAD). The mobile phases A and B used were an aqueous solution containing 1% glacial acetic acid and a methanol solution, respectively. The elution gradient was set as follows: 0 min 85% of mobile phases A, 0–13 min 45% of mobile phases A, 13–18 min 85% of mobile phases A, and kept at 85% of mobile phases A until the end. The column, detection wavelength and flow rate were 30 °C, 280 nm and 0.8 mL min^−1^, respectively.

### 2.10. Simulated Digestion Experiments In Vitro

#### 2.10.1. Preparation of Simulated Digestive Solutions

According to the method by Minekus et al. [28] with some with slight modifications, the simulated digestion model in vitro was established.

Simulated salivary fluid (SSF): 1.73 mL of KCl (0.5 M), 0.424 mL of KH_2_PO_4_ (0.5 M), 0.78 mL of NaHCO_3_ (1 M), 0.0573 mL of MgCl_2_(H_2_O)_6_ (0.15 M) and 6.9 μL of (NH_4_)_2_CO_3_ (0.5 M) were mixed to prepare an oral electrolyte solution, and then 60 μL of α-amylase (15 kU mL^−1^) and 30 μL of CaCl_2_(H_2_O)_2_ (0.3 M) were added for later use.

Simulated gastric fluid (SGF): 1.29 mL of KCl (0.5 M), 0.168 mL of KH_2_PO_4_ (0.5 M), 2.34 mL of NaHCO_3_ (1 M), 2.2 mL of NaCl (2 M), 0.075 mL of MgCl_2_(H_2_O)_6_ (0.15 M) and 0.093 mL of (NH_4_)_2_CO_3_ (0.5 M) were mixed to prepare a gastric electrolyte solution, and then 80 μL of pepsin (300 kU mL^−1^) and 6 μL of CaCl_2_(H_2_O)_2_ (0.3 M) were added for later use.

Simulated intestinal fluid (SIF): 1.34 mL of KCl (0.5 M), 0.158 mL of KH_2_PO_4_ (0.5 M), 8.4 mL of NaHCO_3_ (1 M), 1.9 mL of NaCl (2 M) and 0.217 mL of MgCl_2_(H_2_O)_6_ (0.15 M) were mixed to prepare an intestinal electrolyte solution, and then 96 μL of trypsin (25 kU mL^−1^), 240 μL of bile salt (540 mg mL^−1^) and 48 μL of CaCl_2_(H_2_O)_2_ (0.3 M) were added for later use.

#### 2.10.2. In Vitro Simulated Digestion

Referring to the results of the study reported by Kaur et al. [29], the concentrations of ECG used would only exhibit a very low cytotoxicity. The untreated, heated (100 °C, 20 min) and microwaved (800 W, 6 min) ECG solutions were mixed with equal volumes of SSF, the pH values of the digestive solutions were adjusted to 7.0 by HCl and NaOH, then the samples were digested in an oscillating incubator (37 °C, 100 rpm) for 5 min. The samples after oral digestion were added into SGF, the pH value was adjusted to 2.0, then the simulated gastric digestion was performed for 2 h under the same conditions. Whereafter simulated intestinal digestion was conducted at a pH of 7.0 for 4 h. After each step of digestion, the samples were immediately soaked in ice water to stop the reaction, and then all solutions were frozen at −20 °C.

#### 2.10.3. Assays of Bioaccessibility and Biologic Activity

The ECG standard solutions were prepared, and a standard curve was established by HPLC for quantitative determination of ECG. The bioaccessibility and gastrointestinal digestive stability of ECG can be assessed by the simulating digestion in vitro, and the calculation equations were as follows: Relative bioaccessibility (%) = The content of ECG after intestinal digestion/Raw content of ECG × 100; Absolute bioaccessibility (mM) = The content of ECG after intestinal digestion (mM). The DPPH free radical scavenging activity and the inhibitory activity against XO of ECG samples after simulated digestion in vitro were then determined.

### 2.11. Statistical Analysis

All experiments were performed in triplicate and results were shown as mean ± standard deviation. Statistical analysis was performed using the general linear model procedure of the statistical package for the social sciences 20.0 (SPSS, Chicago, IL, USA) software. Data were analyzed with one-way analysis of variance, and the least significance difference method was used for mean comparison test, and the differences were statistically significant at *p* < 0.05. All graphs were drawn by Origin 8.0 software.

## 3. Results and Discussion

### 3.1. Effects of pH on the Activity of ECG

#### 3.1.1. UV–Vis Absorption Spectra of ECG at Different pH Values

**Figure 2:**
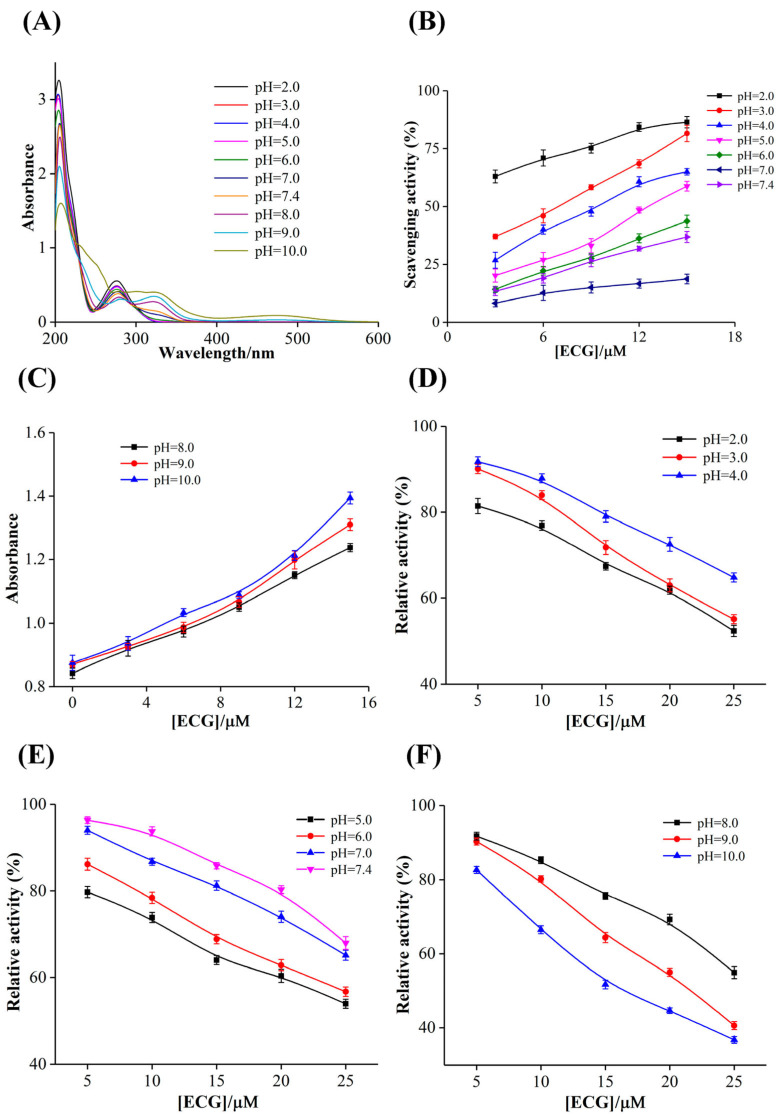
(**A**) UV–vis absorption spectra of ECG at different pH values; *c*(ECG) = 25 μM; (**B**) the scavenging activity of ECG on DPPH radical under acidic conditions; (**C**) absorbance of ECG at 517 nm under alkalic conditions; inhibitory activity of ECG on XO at lower pH (**D**), intermediate pH (**E**) and higher (**F**) pH.

The adjustment of pH value is a common flavoring and processing method in the food industry, as it could improve the processing technology and inhibit microbial growth, thus enhancing the stability and safety of food. However, pH value is also an important factor affecting the stability of active ingredients, and the unsuitable pH may lead to the degradation of polyphenols and reduce their physiological activities. The UV–vis absorption spectra could reflect the changes in the structure and content of substances, which was used to analyze the stability of ECG at different pH values. As shown in Figure 2A, ECG is a weakly acidic phenolic acid, which has higher stability under acidic conditions. When the pH value was lower than 7.0, the sharp absorption peak at 205 nm was caused by the conjugation of benzene and carbonyl group, which was related to the transition of *π* → π* and the charge transfer of *n* → σ*. However, the characteristic absorption peak at 275 nm was caused by other non-conjugated chromophores. It has previously been reported that phenolics are more stable at low pH and that they can auto-oxidize under alkaline conditions, which may lead to formation of coupling products [30]. With the increase in pH value, the intensity of characteristic peaks at 205 nm and 275 nm gradually decreased and weakened, respectively, and the peak positions appeared obvious red shift. When the pH value was higher than 9.0, the characteristic peak at 275 nm disappeared, but a new absorption peak gradually appeared at 325 nm. These findings indicated that ECG was only relatively stable under acidic conditions, and the structure of ECG changed significantly, which was rapidly oxidized and degraded with the increase in pH value.

#### 3.1.2. Effects of pH Value on the Free Radical Scavenging Activity of ECG

The scavenging rates of ECG on DPPH free radical at different pH values were measured, which were divided into two pH ranges to discuss. As the increase in pH value, the scavenging rate of ECG on DPPH radical decreased significantly (Figure 2B), which may be because the stability of ECG was decreased. The increase in pH value would accelerate the oxidation, isomerization and hydrolysis of ECG, which may eventually lead to the decrease in antioxidant capacity. As shown in Figure 2C, when the pH value was above 7.4, the color of the samples became darker with the increase in ECG concentration, and the absorbance values gradually increased, indicating that ECG was rapidly oxidized and degraded under alkaline conditions, and the samples almost lost the scavenging ability on DPPH radical. It was also reported that catechins may produce quinone, superoxide groups and peroxide free radicals, etc., under alkaline conditions and result in oxidative stress [31]. These products may also have characteristic absorption peaks around 517 nm, thus the absorbance values were increased. In summary, ECG had better antioxidant activity in the pH range of 2.0–6.0, indicating that acidic conditions were conducive to the stability of ECG and could slow the oxidation and degradation of ECG to a certain extent. Therefore, it is necessary to maintain a weakly acidic environment to improve the stability of catechin-rich products in the food processing and storage.

#### 3.1.3. Effects of pH Value on the Inhibitory Activity of ECG against XO

The inhibitory activity of ECG on XO after incubation at different pH values was determined. As shown in Figure 2D, ECG better inhibited the activity of XO in the lower pH range (2.0–4.0), and the inhibitory ability decreased with the increase in pH value. The similar results were observed under moderate pH conditions (5.0–7.4), and the higher pH values were not favorable for ECG to exert its inhibitory activity against XO (Figure 2E). The inhibitory activity of ECG against XO increased gradually under alkaline conditions (Figure 2F). Overall, the better inhibitory activity of ECG in acidic conditions may be because it was relatively stable under acidic conditions. In contrast, ECG was gradually oxidized as the increase in pH, thus the inhibitory ability of ECG under neutral conditions was weaker than that under acidic conditions. However, the relative activity of XO decreased to 36% at pH 10.0, which may be due to the drastic oxidation and degradation of ECG at high pH, or the isomerization reaction of ECG and its conversion into other catechins. These products may also have the inhibitory activity on XO, and the various components may show a synergistic inhibitory effect. In a word, different pH values significantly affected the inhibitory activity of ECG on XO, and relatively high inhibitory activity was shown under alkaline conditions.

### 3.2. Effects of Conventional and Microwave Heating Treatments on the Free Radical Scavenging Activity of ECG

#### 3.2.1. Effects of Heating Temperature and Time

**Figure 3:**
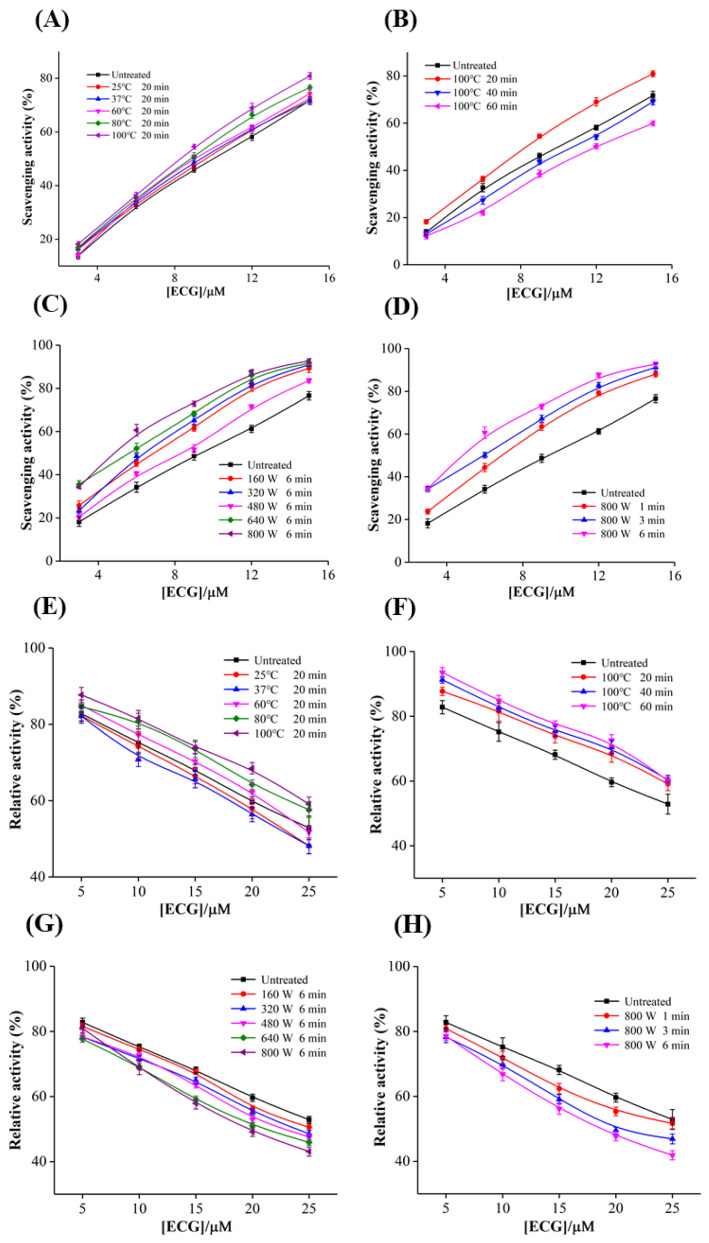
The influence of heating temperature (**A**) and time (**B**) on the DPPH radical scavenging activity of ECG; the influence of microwave power (**C**) and time (**D**) on the DPPH radical scavenging activity of ECG; the influence of heating temperature (**E**) and time (**F**) on the inhibitory activity of ECG against XO; the influence of microwave power (**G**) and time (**H**) on the inhibitory activity of ECG against XO.

As shown in Figure 3A, ECG generally had a certain radical scavenging activity after heating at 25−100 °C. The scavenging ability of ECG on DPPH radical was slightly enhanced with the increase in heating temperature, and the scavenging rate increased from 71.75% to 80.88% at the heating temperature of 100 °C, suggesting that appropriate heating was beneficial to the antioxidant effect of ECG. In addition, the scavenging ability increased when heating for 20 min, but decreased significantly when the heating time exceeded 40 min (Figure 3B). When the concentration of ECG was 15.0 μM, the corresponding scavenging rate decreased from 71.75% to 59.89% after heating at 100 °C for 60 min, which may be because prolonged heating resulted in the continued degradation of ECG, eventually the antioxidant activity was decreased. These results suggested that appropriate heating time and temperature can improve the antioxidant activity of ECG. Park et al. [32] reported the similar effects of heating time and temperature on the antioxidant activity of phenolic substances in turmeric extract. The reasons for the improvement on the antioxidant activity of ECG by heating treatment may be as follows: (I) As a flavanol substance, the solubility of ECG is highly dependent on temperature and solvent. Appropriate heating may increase the solubility of ECG and improve the availability of the sample solution, and thus the antioxidant activity of the system would be enhanced. Zhang et al. [33] also reported that heating increased the antioxidant capacity of blueberry pulp, which could be attributed to the increase in solubility and content of anthocyanin in juice. (II) Catechins may undergo various reactions, such as oxidative, degradation, polymerization, hydrolysis and isomerization at the same time under different environmental and processing conditions. Pineiro, Palma and Barroso [34] reported that catechins mainly underwent isomerization reaction at the temperatures lower than 130 °C, thus may be decomposed into non-phenotypic catechins or hydrolyzed into other phenolic acids with low molecular weights, and these degradation products may also scavenge the free radical in the system. (III) It was found that the color of the ECG samples gradually changed to brown when heating temperature and time were increased to 60 °C and 40 min, respectively, indicating that ECG underwent oxidation and polymerization reactions, and colored substances were generated [35]. Some oxidation products may also have biological activities. In general, although high temperature may affect the stability of ECG, it can also improve its antioxidant activity to a certain extent. In the future, it is necessary to further optimize the thermal processing technology of catechins to ensure the maximum quality of tea products.

#### 3.2.2. Effects of Microwave Power and Time

With the advantages of high efficiency and energy saving, microwave heating has a wide range of applications in domestic, commercial and industrial food processing, such as cooking, drying and sterilization. Microwave technology is likely to be involved in the processing of tea and various foods rich in catechins, thus it is of great significance to explore the effects of microwave treatment on the stability and antioxidant and XO inhibitory activities of catechins. It was observed that the scavenging ability of ECG generally increased with the rise in microwave power within a certain range (Figure 3C). When the concentration was 15.0 μM, the scavenging rate of ECG increased by 20.96% after microwaving at 800 W for 6 min, suggesting that microwave pretreatment significantly improved the antioxidant activity of ECG. The gradual enhanced DPPH free radical scavenging ability of ECG was also found with the prolongation of microwave time (Figure 3D). However, based on the consideration of microwave heating time in daily cooking, 6 min was selected as the microwave processing time for subsequent experiments. These results indicated that microwave treatment can also promote the antioxidant activity of ECG. Similar to conventional heating, the enhanced free radical scavenging activity of ECG by microwave heating could also be attributed to the improvement of solubility, the isomerization reaction and the formation of oxidation products. Compared with traditional heating, the effect of microwave treatment on enhancing the antioxidant activity of ECG was more significant, indicating that microwave heating can be used as an effective approach to enhance the antioxidant activity of catechins. Martins et al. [36] explored the effects of microwave heating on the biological activities of orange juice, and found that orange juice had higher antioxidant activity and stronger inhibitory activities on α-amylase and α-glucosidase after appropriate microwave treatment.

### 3.3. Effects of Conventional and Microwave Heating Treatments on the Inhibitory Activity of ECG against XO

#### 3.3.1. Effects of Heating Temperature and Time

As shown in Figure 3E, with the increase in heating temperature, the inhibitory activity of ECG on XO tended to increase first and then decrease. Untreated ECG reduced the activity of XO from 82.81% to 52.89%, and the heating treatments at 25 °C and 37 °C reduced the activity of XO to 48.15%. When the heating temperature reached 60 °C, the inhibitory activity decreased significantly with the increase in temperature. After heating at 100 °C, ECG only reduced the relative activity of XO to 59.09% at a concentration of 25.0 μM. As shown in Figure 3F, with the prolongation of heating time, the inhibitory ability of ECG on XO gradually weakened, and ECG only inhibited 40.87% of the XO activity when heated at 100 °C for 60 min. These results indicate that lower heating temperature and shorter heating time could enhance the inhibitory ability of ECG against XO, while prolonged high-temperature heating would lead to a significant decrease in its inhibitory activity. It has been reported that moderate processing temperatures were favorable for the oxidation and isomerization of catechins in tea beverages, while too high temperatures could lead to significant degradation [37]. Thus, it can be inferred that the solubility of ECG under appropriate heating conditions increased, and a small part of ECG underwent isomerization reaction and was converted into other active components, which may improve the antioxidant and XO inhibitory activity of ECG, but most ECG has been degraded at high temperatures, thus the inhibitory capability decreased sharply.

#### 3.3.2. Effects of Microwave Power and Time

As shown in Figure 3G, with the increase in microwave power within a certain range, the inhibitory ability of ECG on XO was gradually enhanced, and the relative activity of XO decreased to 43.11% at a treatment power of 800 W. In addition, the increase in microwave treatment time also promoted the inhibition of XO by ECG (Figure 3H). When the concentration reached 25.0 μM, the inhibitory rate under microwave treatment for 6 min at a power of 800 W (56.89%) was significantly higher than the untreated sample (47.11%), indicating that ECG treated by microwave could effectively enhance its inhibitory activity against XO. Microwave heating is a highly efficient thermal processing technology, thus its mechanism of improving antioxidant and XO inhibitory activity of ECG was similar to conventional heating treatment. Compared with conventional heating, microwave treatment had a more significant improvement in the antioxidant and XO inhibitory activity. Moreover, microwave heating has the advantages of high efficiency and low energy consumption. It has also been reported [38] that microwave can better maintain the original color and stability of food components. Therefore, in the processing of food rich in catechins, microwave heating can be considered instead of traditional heating, which is conducive to ensure the quality and improve the functional activity of products.

### 3.4. Analysis of Binding Properties

**Figure 4:**
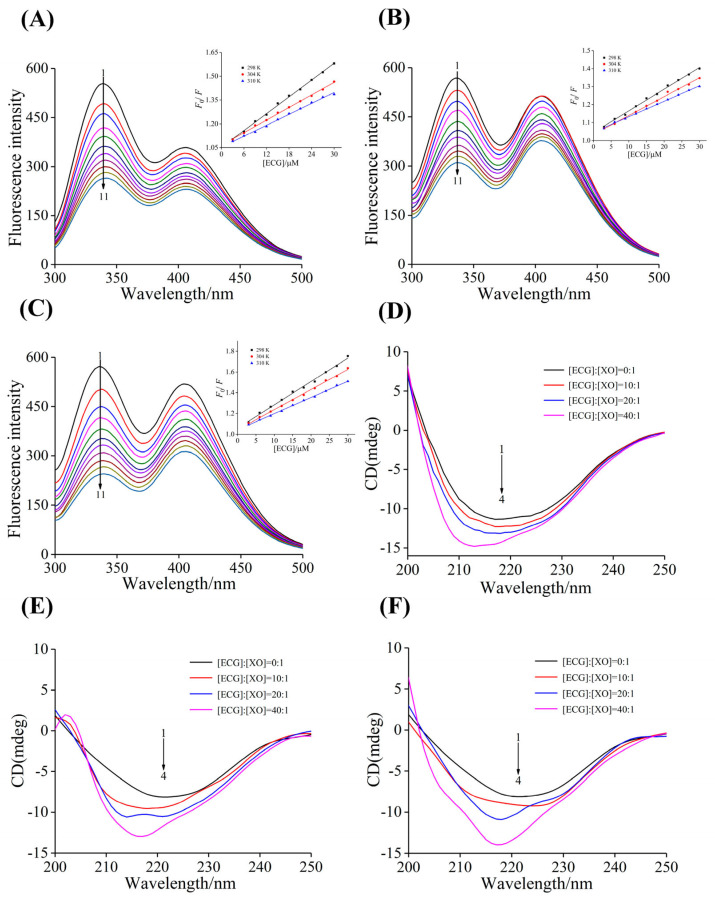
Fluorescence quenching and Stern–Volmer plots of XO by ECG after untreated (**A**), heated (100 °C, 20 min) (**B**) and microwaved (800 W, 6 min) (**C**); *c*(XO) = 1.0 μM, *c*(ECG) = 0, 3.0, 6.0, 9.0, 12.0, 15.0, 18.0, 21.0, 24.0, 27.0 and 30.0 μM for Curves 1 → 11, respectively; Effects of ECG on the CD spectra of XO after untreated (**D**), heated (100 °C, 20 min) (**E**) and microwaved (800 W, 6 min) (**F**); *c*(XO) = 1.0 μM, the molar ratios of ECG to XO were 0:1, 10:1, 20:1 and 40:1 for Curves 1 → 4, respectively.

Inhibitors mainly inhibit the enzyme activity by interacting with enzyme and changing the structure of enzyme, thereby preventing the normal reaction between enzyme and substrate. Therefore, in order to explore the underlying mechanism of the effects of heating and microwave treatments on the inhibitory effect of ECG against XO, the fluorescence quenching experiments were carried out and the changes in the binding properties of ECG and XO before and after treatments were analyzed. As shown in Figure 4A–C, the untreated, heated and microwaved ECG samples significantly quenched the endogenous fluorescence of XO, and the shape and position of the emission spectra remained basically unchanged, indicating that ECG still bound to XO in a non-covalent manner. The *F*_0_/*F* curve of ECG treated by heating and microwave showed good linearity, and the curve slope *K*_sv_ values increased with the decrease in temperature, and the *K*_q_ values were much higher than the maximum scattering collision quenching rate constant (2.0 × 10^10^ L mol^−1^ s^−1^) (Table 1, Figure 4A–C). These results showed that heating and microwave treatments did not affect the fluorescence quenching type of ECG on XO, which was still a single static quenching.

Compared with untreated ECG, the *K*_sv_ values of ECG after heating treatment at 100 °C decreased, and the *K*_a_ values also decreased from (1.82 ± 0.05) to (0.92 ± 0.15) × 10^4^ L mol^−1^. On the contrary, the results of microwave treatment showed that both *K*_sv_ and *K*_a_ increased, and *K*_a_ values increased from (1.82 ± 0.05) to (2.18 ± 0.01) × 10^4^ L mol^−1^, indicating that high heating temperature was not conducive to the binding of ECG and XO, while microwave treatment can enhance the fluorescence quenching ability and the binding affinity of ECG to XO. Therefore, conventional high temperature heating and microwave treatment weakened and enhanced the inhibitory ability of ECG against XO, respectively. Yu et al. [15] also reported that microwave treatment enhanced the binding of asparagus polyphenols with tyrosinase and improved its anti-browning ability. The *n* values were close to 1 before and after treatment, meaning that there was only one type of binding site for ECG in the XO molecule.

The results of thermodynamic analysis (Table 1) showed that the Δ*H*°, Δ*S*° and Δ*G*° values of the binding between ECG and XO before and after heating and microwave treatments were all negative, suggesting that the binding driving forces were hydrogen bond and van der Waals force. Under the two processing conditions, the values of Δ*H*° and Δ*S*° were significantly decreased, indicating that the thermal stability of the complexes formed after treatments increased and the disorder of the systems decreased.

### 3.5. Analysis of Secondary Structure

As shown in Figure 4D–F, after heating and microwave treatments, the signal of the CD spectral peak gradually increased with the increase in ECG concentration, and the obvious changes in shape and position of the characteristic peak were observed. There was a large blue shift in the peak position, indicating that the effect of ECG on the structure of XO changed after heating and microwave treatments. As shown in Table 2, the addition of untreated ECG led to an increase in the contents of α-helix and β-sheet of XO, while the contents of β-turn and random coil were decreased, indicating that ECG induced the contraction of structure and the enhancement of rigidity for XO.

In contrast, the α-helix contents of XO were increased by 40.67%, 50.18% and 60.76% in the untreated, heating and microwaved ECG samples, respectively, indicating that the heating and microwave treatments induced a greater increase in the α-helix content of XO. However, the β-sheet contents of XO in the treated ECG samples were decreased by 10.27% and 9.67%, respectively. These results suggest that the two processing methods strengthened the influence of ECG on the secondary structure of XO, and the binding of ECG with XO was improved, leading to a greater increase in the structural compactness of XO and a decrease in molecular flexibility, thereby hindering the entry of substrate and the release of the catalytic products, eventually increasing the inhibitory activity of ECG against XO. In addition, the effects of microwaved ECG on the structure of XO were more significant than that of traditional heating, which further explained the result that microwave treatment had a more pronounced effect on the antioxidant and XO inhibitory activity of ECG.

### 3.6. Degradation Mechanism of ECG

**Figure 5:**
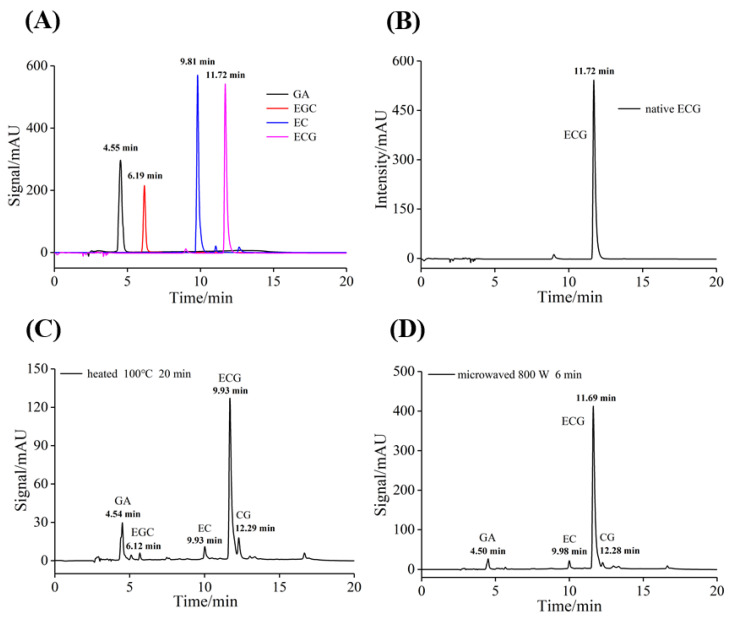
HPLC chromatograms of standard samples (**A**), untreated (**B**), heated (100 °C, 20 min) (**C**) and microwaved (800 W, 6 min) (**D**) ECG solutions; *c*(ECG/GA/EGCG/EGC) = 1.0 mM.

HPLC technique was used to separate and identify the ECG systems after heating and microwave treatments, which aimed to determine the isomerization and degradation products of ECG and further elaborate the mechanism of the bioactivity enhancement of ECG by these two treatments. The characteristic peaks of several standard catechins showed HPLC had an excellent separation effect (Figure 5A). As shown in Figure 5B, the characteristic peak of untreated ECG appeared at the retention time of 10.71 min, while new absorption peaks appeared in the chromatogram of ECG after heating and microwave treatments (Figure 5C,D), indicating that ECG has been transformed and degraded.

Based on the results, it could be inferred that heating at 100 °C for 20 min may lead to the following changes in ECG: (I) ECG isomerized to form epimer CG; (II) ECG produced EGC by the hydrolysis and hydroxylation process, and then EC was formed via the hydrolysis of EGC; (III) ECG and CG removed gallic groups to form gallic acid (GA) and EC. The chromatographic peak at the elution time of 16.65 min may be the absorption peak of some oxidation products from ECG. A similar transformation mechanism occurred in the microwaved ECG sample, which degraded to GA, CG and EC.

Figure 5 showed the peak height of ECG decreased significantly after conventional heating and microwave treatment, suggesting that a large degradation reaction occurred in the ECG system. As shown in Table 3, heating and microwave treatment led to the decrease in the ECG content by 33% and 13%, respectively. The changes in ECG caused by microwave treatment were smaller, indicating that the influence of conventional heating on the stability of ECG was higher than that of microwave treatment. In addition, compared with other degradation reactions, the ECG treated with heating and microwave mainly underwent the reaction of removing gallic group, which may be due to the relatively high stability of GA, thus the content of GA in the solutions gradually increased. Therefore, it could be deduced that the enhanced antioxidant and XO inhibitory activity of ECG by the two processing methods may be because a small part of GA and other catechins were produced in the ECG system. It has been also reported [39] that the presence of GA enhanced the ability of phenolic substances to chelate metal ions, thereby enhancing the antioxidant activity of polyphenols. In addition, these reaction products may also have antioxidant and XO inhibitory activity [40], and may also have synergistic effects with ECG, thus the antioxidant and XO inhibitory activity of ECG have been improved by the processing treatments. Similar results were reported by Hsieh et al. [41] who found that the inhibitory activity of bitter melon on α-glucosidase was enhanced by heating treatment due to the formation of Maillard reaction products.

### 3.7. Bioaccessibility, Antioxidant and XO Inhibitory Activity of ECG after Simulated Digestion

#### 3.7.1. Bioaccessibility of ECG

**Figure 6:**
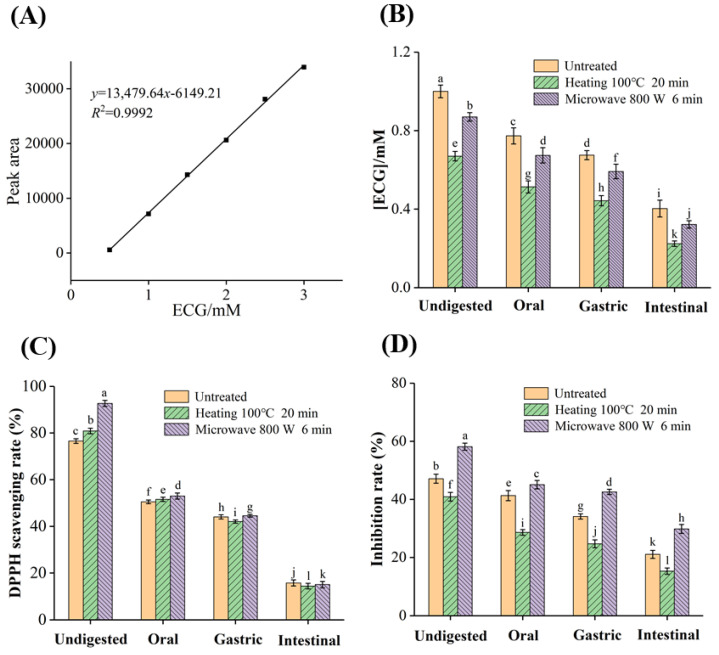
(**A**) The standard curve of ECG; (**B**) the concentration changes in untreated, heated (100 °C, 20 min) and microwaved (800 W, 6 min) ECG during simulated digestion; the scavenging rate on DPPH free radical (**C**) and the inhibitory rate on XO (**D**) of ECG before and after simulated digestion. The least significance difference method was used for mean comparison test according to column and the different letters indicate a significant difference (*p* < 0.05).

As shown in Figure 6A,B, and the content of ECG samples decreased significantly after digestion. The concentration of untreated ECG (1.00 mM) was decreased to 0.67 mM and 0.87 mM after heating and microwave treatments, respectively, while the concentrations of the three groups of samples were decreased to 0.40 mM, 0.22 mM and 0.32 mM after simulated oral, gastric and intestinal digestion, respectively (Table 3). The bioaccessibility of untreated ECG (40.30%) was decreased to 32.84% and 36.78% after heating and microwave treatments, respectively. The decreased content of ECG after digestion may be because it interacted with the digestive enzymes (α-amylase, pepsin and trypsin), and the hydroxyl groups in ECG were connected with the enzymes through hydrogen bonds to form macromolecular polymers [42]. In contrast, the contents of ECG in the three groups of samples were significantly lower in the gastric digestion stage than in oral and intestinal digestion. This may be because ECG was a weakly acidic substance, and was relatively stable in the acidic environment of SGF, while the degradation and oxidation easily occurred in the neutral SIF. In conclusion, the presence of digestive enzymes and the pH value of the digestive fluids were the key factors affecting the gastrointestinal stability of ECG, which was consistent with the results of the effects of pH on the antioxidant and XO inhibitory activity of ECG.

In addition, heating and microwave treatments further decreased the bioaccessibility of ECG, and the possible reasons may be as follows: (I) the conformational stability of ECG after processing was reduced, and it was more prone to degradation and oxidation; (2) the presence of GA, EGC, EC, etc., promoted the binding of ECG with digestive enzymes. Moreover, the bioaccessibility of ECG was higher under microwave treatment, indicating that the microwave treatment could reduce the loss of catechins compared with conventional heating, which was a green and efficient food processing method.

#### 3.7.2. Effects of Simulated Digestion on the Antioxidant Activity of ECG

Before digestion, heating and microwave treatments enhanced the radical scavenging activity of ECG, while the scavenging rate was greatly reduced and the difference in scavenging ability of the three groups of samples was reduced after oral digestion (Figure 6C). The scavenging rates in untreated, heating and microwaved ECG decreased from 76.56%, 80.88% and 92.71% to 15.85%, 14.48% and 15.12%, respectively. It was worth noting that the radical scavenging rate of the three groups of samples in the oral and intestinal digestion stages decreased greatly, which was consistent with the decreasing trend of concentration of ECG. The difference in the radical scavenging activity of the three groups of samples was decreased after simulated digestion, and the scavenging rate of ECG after heating treatment was the lowest, indicating that simulated digestion may weaken the effects of the two processing methods on the antioxidant activity of ECG [43]. In comparison, the antioxidant effect of microwave-treated ECG after digestion was still better than that of conventional heating, possibly due to the higher digestion stability of ECG under microwave treatment.

#### 3.7.3. Effects of Simulated Digestion on the Inhibitory Activity of ECG against XO

As shown in Figure 6D, the ECG after microwave treatment had the highest inhibitory rate in the undigested samples. After each of digestion stage, the inhibitory ability of ECG on XO gradually decreased. At last, the inhibitory rates of untreated, heating and microwaved ECG samples against XO decreased from 47.11%, 40.90% and 58.14% to 21.06%, 15.30% and 29.80%, respectively, which was mainly due to the reduced ECG content. The inhibitory ability of the three groups of samples on XO was in the same order before and after digestion, which may be related to the degradation of ECG. Microwave treatment converted part of ECG into active components, such as EGC, EC and GA. The coexistence of these degradative products enhanced the interaction of ECG with XO, thus exhibiting the synergistic inhibitory effects. However, conventional heating led to substantial degradation and oxidation of ECG, resulting in a sharp decrease in the content, thus weakening the inhibitory ability. These findings further demonstrated that microwave treatment was a mild and efficient food processing technology, and may induce appropriate degradation of ECG, thus enhancing its antioxidant and XO inhibitory activity [44].

## 4. Conclusions

The results of this work showed that ECG was relatively stable under acidic conditions, and had higher biological activity. Microwave treatment significantly improved ECG’s scavenging ability on DPPH radicals and the inhibition of XO, and its effects were better than conventional heating. The mechanism by which microwave treatment enhanced the inhibitory ability of ECG against XO was to increase the binding affinity and strengthen the influences of ECG on the secondary structure of XO. ECG was degraded into CG, EGC, EC and GA after heating and microwave treatments. Compared with conventional heating, microwave treatment resulted in higher gastrointestinal stability and biological activity of ECG. Hence, microwave heating was a green, mild and efficient food processing method that could effectively degrade catechins moderately to enhance their biological activity. These findings have provided the basic data for the rational development and utilization of catechins. In the future, the effects of a combination of multiple processing methods on food components need to be investigated.

## Figures and Tables

**Table 1:** The values of *K*_sv_, *K*_a_, *n* and thermodynamic parameters for the binding of XO with ECG under different treatment conditions.

Sample	*T* (K)	*K*_sv_ (×10^4^ L mol^−1^)	*R* ^a^	*K*_a_ (×10^4^ L mol^−1^)	*R* ^b^	*n*	Δ*H*° (kJ mol^−1^)	Δ*G*° (kJ mol^−1^)	Δ*S* (J mol^−1^ K^−1^)
Untreated	298	1.76 ± 0.02 ^c^	0.9982	1.82 ± 0.05 ^b^	0.9934	0.91 ± 0.01	−25.05 ± 0.20	−24.32 ± 0.40	−2.44 ± 0.07
	304	1.32 ± 0.03 ^e^	0.9984	1.51 ± 0.02 ^d^	0.9928	0.95 ± 0.02		−24.31 ± 0.35	
	310	1.15 ± 0.01 ^g^	0.9964	1.23 ± 0.04 ^f^	0.9985	0.87 ± 0.02		−24.29 ± 0.28	
Heating (100 °C, 20 min)	298	1.21 ± 0.02 ^f^	0.9963	0.92 ± 0.12 ^g^	0.9911	0.89 ± 0.02	−37.70 ± 0.32	−22.69 ± 0.71	−50.38 ± 1.05
	304	1.05 ± 0.04 ^h^	0.9950	0.74 ± 0.17 ^h^	0.9808	0.84 ± 0.03		−22.38 ± 0.80	
	310	0.86 ± 0.01 ^i^	0.9984	0.51 ± 0.11 ^i^	0.9897	0.79 ± 0.02		−22.08 ± 0.55	
Microwave (800 W, 6 min)	298	2.25 ± 0.05 ^a^	0.9948	2.18 ± 0.01 ^a^	0.9944	0.88 ± 0.02	−34.57 ± 0.11	−24.78 ± 0.86	−32.85 ± 0.34
	304	1.94 ± 0.03 ^b^	0.9973	1.70 ± 0.02 ^c^	0.9882	0.90 ± 0.03		−24.58 ± 0.64	
	310	1.56 ± 0.02 ^d^	0.9984	1.27 ± 0.03 ^e^	0.9870	0.87 ± 0.03		−24.39 ± 0.53	

*R*^a^ and *R* ^b^ are the correlation coefficients for *K*_SV_ and *K*_a_ values, respectively. The least significance difference method was used for mean comparison test according to column and the different letters indicate a significant difference (*p* < 0.05).

**Table 2:** Effects of untreated, heated (100 °C, 20 min) and microwaved (800 W, 6 min) ECG on the secondary structure of XO.

Sample	Molar Ratio [ECG]:[XO]	α−Helix (%)	β−Sheet (%)	β−Turn (%)	Random Coil (%)
Free XO	0:1	8.41 ± 0.21 ^j^	41.59 ± 0.36 ^d^	22.27 ± 0.36 ^a^	27.73 ± 0.36 ^g^
ECG–XO (Untreated)	10:1	10.83 ± 0.51 ^f^	42.51 ± 0.41 ^c^	21.65 ± 0.94 ^d^	25.12 ± 0.69 ^h^
	20:1	10.96 ± 0.62 ^e^	43.41 ± 0.74 ^b^	21.59 ± 0.13 ^e^	23.03 ± 0.31 ^i^
	40:1	11.83 ± 0.93 ^c^	45.02 ± 0.22 ^a^	21.39 ± 0.25 ^g^	22.51 ± 0.24 ^j^
ECG–XO (Heating) (100 °C, 20 min)	10:1	8.66 ± 0.34 ^i^	40.74 ± 0.24 ^e^	21.91 ± 0.18 ^b^	28.69 ± 0.11 ^e^
	20:1	10.98 ± 0.74 ^d^	38.08 ± 0.19 ^h^	21.76 ± 0.14 ^c^	29.17 ± 0.43 ^b^
	40:1	12.63 ± 0.26 ^b^	37.32 ± 0.55 ^j^	20.47 ± 0.31 ^h^	29.58 ± 0.38 ^a^
ECG–XO (Microwave) (800 W, 6 min)	10:1	9.18 ± 0.19 ^h^	40.52 ± 0.46 ^f^	21.76 ± 0.47 ^c^	28.54 ± 0.71 ^f^
	20:1	10.38 ± 0.65 ^g^	39.33 ± 0.75 ^g^	21.54 ± 0.19 ^f^	28.75 ± 0.82 ^d^
	40:1	13.52 ± 0.62 ^a^	37.57 ± 0.91 ^i^	20.08 ± 0.54 ^i^	28.93 ± 0.68 ^c^

The least significance difference method was used for mean comparison test according to column and the different letters indicate a significant difference (*p* < 0.05).

**Table 3:** Concentration changes in untreated, heated (100 °C for 20 min) and microwaved (800 W for 6 min) ECG during simulated digestion.

ECG	Concentration (mM)				Bioaccessibility (%)
	Undigested	Oral	Gastric	Intestinal	
**Untreated**	1.00 ± 0.03 ^a^	0.77 ± 0.04 ^c^	0.68 ± 0.02 ^d^	0.40 ± 0.04 ^i^	40.30 ± 2.71 ^A^
Heating (100 °C, 20 min)	0.67 ± 0.02 ^e^	0.51 ± 0.03 ^g^	0.44 ± 0.03 ^h^	0.22 ± 0.01 ^k^	32.84 ± 0.49 ^C^
Microwave (800 W, 6 min)	0.87 ± 0.02 ^b^	0.68 ± 0.04 ^d^	0.59 ± 0.04 ^f^	0.32 ± 0.02 ^j^	36.78 ± 1.42 ^B^

The least significance difference method was used for mean comparison test, and the concentration values were conducted the mean comparison between data from all rows and columns, while the bioaccessibility values were compared according to column. The different letters indicate a significant difference (*p* < 0.05).

## Data Availability

Data is contained within the article.
